# Correction: Molecular predictors of brain metastasis-related microRNAs in lung adenocarcinoma

**DOI:** 10.1371/journal.pgen.1009139

**Published:** 2020-10-14

**Authors:** Guogui Sun, Xiao Ding, Nan Bi, Zhiwu Wang, Lihong Wu, Wei Zhou, Zitong Zhao, Jingbo Wang, Weimin Zhang, Jing Fan, WenJue Zhang, Xin Dong, Ning Lv, Yongmei Song, Qimin Zhan, LuHua Wang

Two of the images in Fig 7F are incorrectly duplicated. The authors have provided a corrected version here.

**Fig 7:**
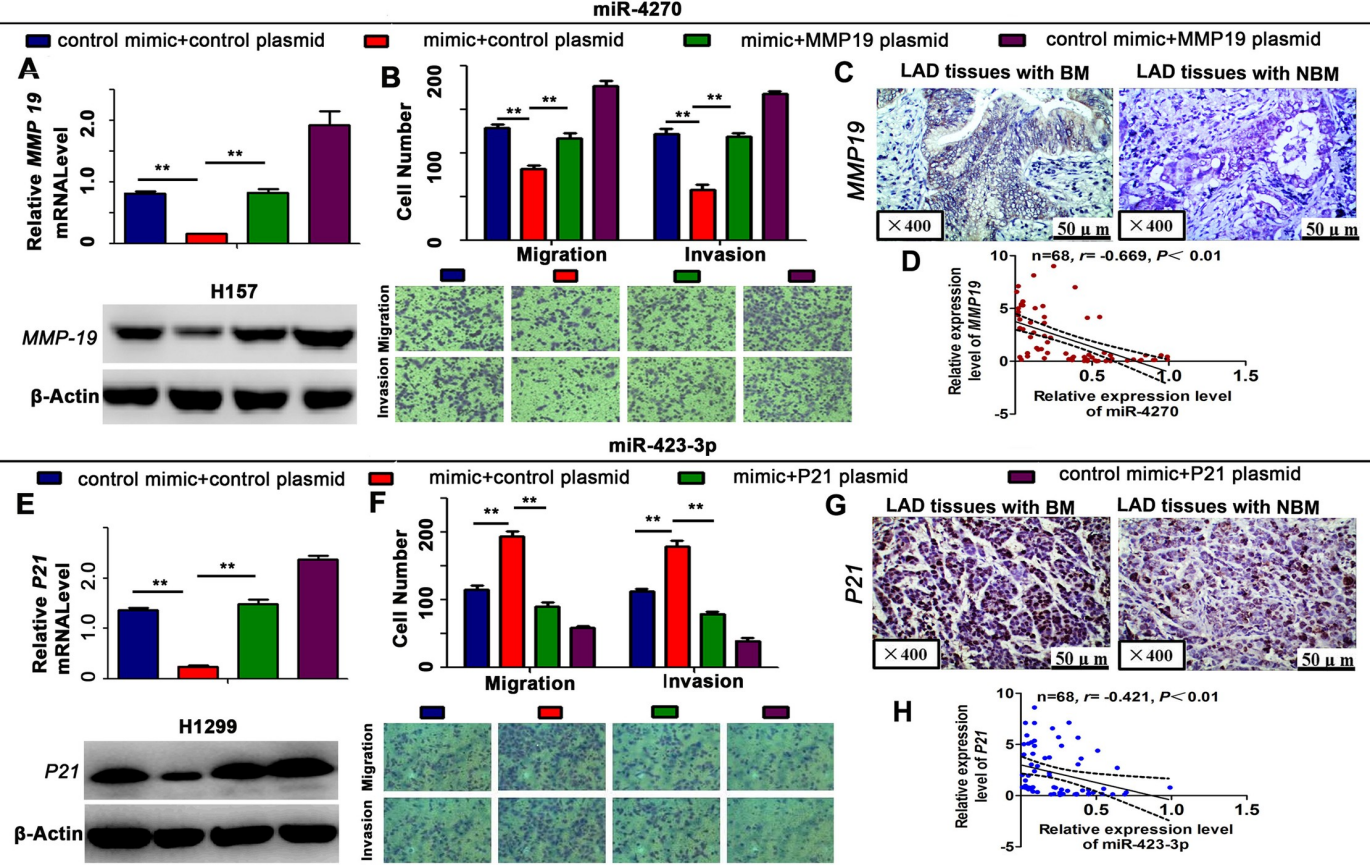
Rescue assay of miR-4270 and miR-423-3p. (A) The mRNA and protein expression levels of *MMP-19*. (B) The results of the migration assay after the co-transfection of cells with the miR-4270 mimic and the pEGFP-C1 plasmid containing an *MMP-19* CDS sequence. (C) *MMP-19* protein expression, as measured by immunohistochemical staining, in LAD samples. (D) Spearman correlation analysis showing the negative correlation between *MMP-19* and miR-4270 expression. (E) The mRNA and protein expression levels of *P21*. (F) The results of the migration assay after the co-transfection of cells with the miR-423-3p mimic and pEGFP-C1 plasmid containing a *P21* CDS sequence. (G) *P21* protein expression, as measured by immunohistochemical staining, in LAD samples. (H) Spearman correlation analysis showing the negative correlation between *P21* and miR-423-3p expression. Data are presented as the means ± SD of three experiments. ** P < 0.01.
